# Factors associated with chronic musculoskeletal pain in Japanese community-dwelling older adults: A cross-sectional study

**DOI:** 10.1097/MD.0000000000007069

**Published:** 2017-06-08

**Authors:** Tatsuya Hirase, Hideki Kataoka, Shigeru Inokuchi, Jiro Nakano, Junya Sakamoto, Minoru Okita

**Affiliations:** a Department of Physical Therapy Science, Nagasaki University Graduate School of Biomedical Sciences; b Department of Locomotive Rehabilitation Science, Nagasaki University Graduate School of Biomedical Sciences, Sakamoto; c Department of Rehabilitation, Nagasaki Memorial Hospital, Hukahori, Nagasaki, Japan.

**Keywords:** aging, catastrophization, chronic pain

## Abstract

Identifying older adults with chronic musculoskeletal pain (CMP) earlier is urgent because CMP is reportedly associated with deterioration in physical function, poor psychological status, and low physical activity level. The objective of this study was to identify factors that were most strongly associated with CMP in Japanese community-dwelling older adults.

Using a cross-sectional design, we assessed 263 older adults (mean age = 79.1 ± 5.9 years, 85.9% women) who participated in community exercise classes. Participants’ physical function, psychological status, and activity levels were evaluated as outcome measures using a variety of tests and instruments. These assessments were conducted prior to beginning the exercise intervention program and compared participants with and without CMP. Additionally, relevant participant characteristics were collected and analyzed. In this study, CMP was defined as the presence of related symptoms within the past month that continued for at least 6 months and corresponded to a numerical rating scale of at least 5 or more at the site of maximum pain.

A total of 143 (54.4%) participants met the criteria for CMP, and a high number of them had chronic lower back pain (64.3%). Outcome measures for the CMP group were significantly worse than for the non-CMP group (*P* < .05). Logistic regression analysis revealed that the Pain Catastrophizing Scale helplessness domain scores (odds ratio: 1.20, 95% confidence interval: 1.09–1.32) with an estimated value of 10 points was the factor most significantly associated with the presence of CMP.

These findings suggest that assessment of the helplessness associated with pain-related catastrophizing is important for identification and the creation of interventions for older adults with CMP.

## 1 Introduction

Chronic musculoskeletal pain (CMP) causes a rise in healthcare costs, deterioration in quality of life, and is a common symptom among community-dwelling older adults.^[1–3]^ Epidemiological studies reveal that the proportion of community-dwelling older adults with CMP ranges from 65.0% to 78.8%, demonstrating its high prevalence.^[2,3]^ Previous studies report that CMP is associated with decline in activities of daily living^[4,5]^ because of deterioration in physical functioning,^[4,6]^ poor psychological status,^[7,8]^ and low physical activity level.^[9,10]^ Therefore, it is urgent to identify older adults with CMP earlier and develop a means of preventing CMP in community-dwelling older adults.

In Japan, the number of community-dwelling older adults that require long-term care due to physical deterioration has increased in recent years as the result of a rapidly aging population.^[11]^ Consequently, to prevent this population from requiring future long-term care, exercise classes are often offered to older adults to improve physical functioning.^[1,11,12]^ Assessments of individuals who participate in these community-based exercise classes have focused on physical functioning, psychological status, and physical activity level. Subsequently, several studies have reported that these classes have effectively improved these outcomes.^[1,11–13]^ However, efforts at preventing CMP are behind compared to Western countries because there is a lack of recognition that CMP is a serious social issue in Japan.^[1,14]^ Therefore, a full pain assessment including factors such as sites, intensity, and time periods has not been conducted for exercise classes, and the characteristics of older adults with CMP remain unclear.

Hasegawa et al^[2]^ reported that a community-based exercise class significantly reduced pain intensity and improved older adults’ physical function. Although our previous study revealed that this class reduced pain intensity and improved physical activity level, these positive effects were only found for individuals without CMP.^[15]^ Therefore, it is important to develop an effective intervention program for community-dwelling older adults that is based on and accounts for the factors most closely associated with CMP. Additionally, the estimated value of the above-mentioned factors to identify older adults with CMP is useful for their early identification.

Research in the field of pain medicine defines pain-related catastrophizing as “an exaggerated negative orientation toward pain stimuli and pain experience” (p. 524).^[16]^ This definition reflects the cognitive aspects of pain, which may also be related to the aforementioned negative effects of CMP.^[17]^ Furthermore, the influence of cognition could be similar for both community-dwelling older adults as well as other patients with CMP. Consequently, we investigated the characteristics of older adults with CMP who participated in community exercise classes to identify the factors that were most strongly related to the presence of CMP. Moreover, we investigated the estimated value of the most related factors to identify older adults with CMP.

## 2 Methods

### 2.1 Design and participants

This cross-sectional study cited the STROBE statement^[18]^ and was conducted in the Japanese city of Unzen from April 2015 to November 2016. A total of 306 older adults participated in the community exercise classes. Participant inclusion criteria were as follows: aged 65 years or older, living at home, able to walk outdoors without a cane, and independence in activities of daily living. Individuals unable to respond to interview questions because of a cognitive impairment were excluded. Persons who had a neurological condition, a cardiovascular condition, or severe arthritis disorder were also excluded. This study was approved by the Nagasaki University Graduate School of Biomedical Sciences and met the guidelines of the 2008 Helsinki Declaration of Human Rights. Written informed consent was obtained from each participant prior to study participation.

Of the 306 potential participants, 37 (12.1%) were excluded because of cognitive impairment, and 6 (2.0%) were excluded for providing incomplete data; therefore, 263 participants (85.9%) met the inclusion criteria and were included in the study.

### 2.2 Assessment

In this study, participants’ pain, physical functioning, psychological status, and physical activity were assessed using a variety of methods prior to beginning the exercise intervention program.

Pain was assessed by determining the total number of pain sites and intensity at the site of maximum pain using a numerical rating scale ranging from 0 (“no pain”) to 10 (“the worst imaginable pain”) as well as the consecutive pain period. Pain assessment was conducted using a body chart. Specifically, participants were asked to identify pain sites and their intensity on the body chart using the scale. Based on this chart, the pain sites were classified into neck, shoulder, upper limb, lumbar (lower back), hip, thigh, knee, and below the knee areas.

Physical functioning was assessed using the following performance tests: the chair stand test (CST)^[19]^ and the timed up-and-go test (TUG).^[20]^ The CST and TUG tests have been identified as markers of lower extremity muscle strength^[19]^ and walking ability,^[20]^ respectively. These tests were conducted twice, and the best values from each of the 2 tests were recorded. Pain and physical functioning assessments were conducted by physical therapists who supported community exercise classes. Physical therapists received training on assessment protocols from one of the study authors prior to study commencement.

Psychological status was evaluated using the 15-item version of the Geriatric Depression Scale (GDS-15),^[21]^ the modified Japanese version of the Falls Efficacy Scale (FES),^[12]^ and the Japanese version of the Pain Catastrophizing Scale (PCS). The PCS consisted of the 13 items developed by Sullivan et al^[16]^ and included 3 domains categorized as rumination (5 items), helplessness (5 items), and magnification (3 items).^[22]^ Each item was rated on a 5-point scale, ranging from 0 (“not at all”) to 4 (“all the time”). Total PCS score ranges from 0 to 52, with higher scores indicating greater pain catastrophizing. Assessments of psychological status were self-administered with guidance from community exercise class staff as required.

Physical activity was measured using a pedometer (Kenz Lifecorder GS, SUZUKEN Co., Ltd, Nagoya, Japan) with an acceleration sensor. Participants were instructed to wear it on their belt or waistband in the right midline of the thigh from the moment they got up until they went to bed, except while bathing or swimming. Participants were also instructed to wear the pedometer for 7 consecutive days prior to beginning the intervention and 7 consecutive days after starting the intervention. Based on Matsubara et al's previous report,^[23]^ we calculated the mean values of participants’ daily step counts and activity times during the wearing period, and categorized them as mild (1–3 metabolic equivalents [Mets]), moderate (4–6 Mets), and heavy (7–9 Mets) to assess the differences in their activity intensity.

### 2.3 Statistical analysis

Statistical analyses were performed using SPSS 22.0 for Windows (SPSS Inc., Armonk, NY). In Nakamura et al^[1]^ previous study, CMP was defined as the presence of related symptoms within the past month that continued for at least 6 months and corresponded to a numerical rating scale of at least 5 or more at the site of maximum pain. Based on this report and using the results of our pain assessment tools, participants who met these criteria were classified as having CMP and denoted the CMP group. Participants who did not meet these criteria were designated the non-CMP group. Unpaired *t* tests were used to evaluate significant differences in age, height, body weight, physical functioning performance tests, and physical activity levels between the 2 groups. Additionally, Mann–Whitney *U* tests were used to assess significant between-group differences in GDS-15 scores, FES scores, and the total PCS score as well scores on each of the 3 PCS domains. Chi-square tests were conducted to compare the sex distribution between the 2 groups. Finally, a logistic regression analysis was performed to identify the factors most strongly associated with the presence of CMP, and receiver operating characteristics (ROC) curves were conducted to identify the estimated value of the selected factors. We calculated the cut-off points, sensitivity, and specificity of the selected factors. A 2-sided *P*-value of ≤.05 was considered statistically significant.

## 3 Results

### 3.1 Participants’ characteristics

The participants’ characteristics are summarized in Table 1. The mean age of the 263 participants was 79.1 (SD = 5.9) years, and 226 (85.9%) were women. Additionally, the mean total number of pain sites and intensity at the site of maximum pain were 2.5 (SD = 1.9) and 4.8 (SD = 2.8), respectively.

**Table 1:**
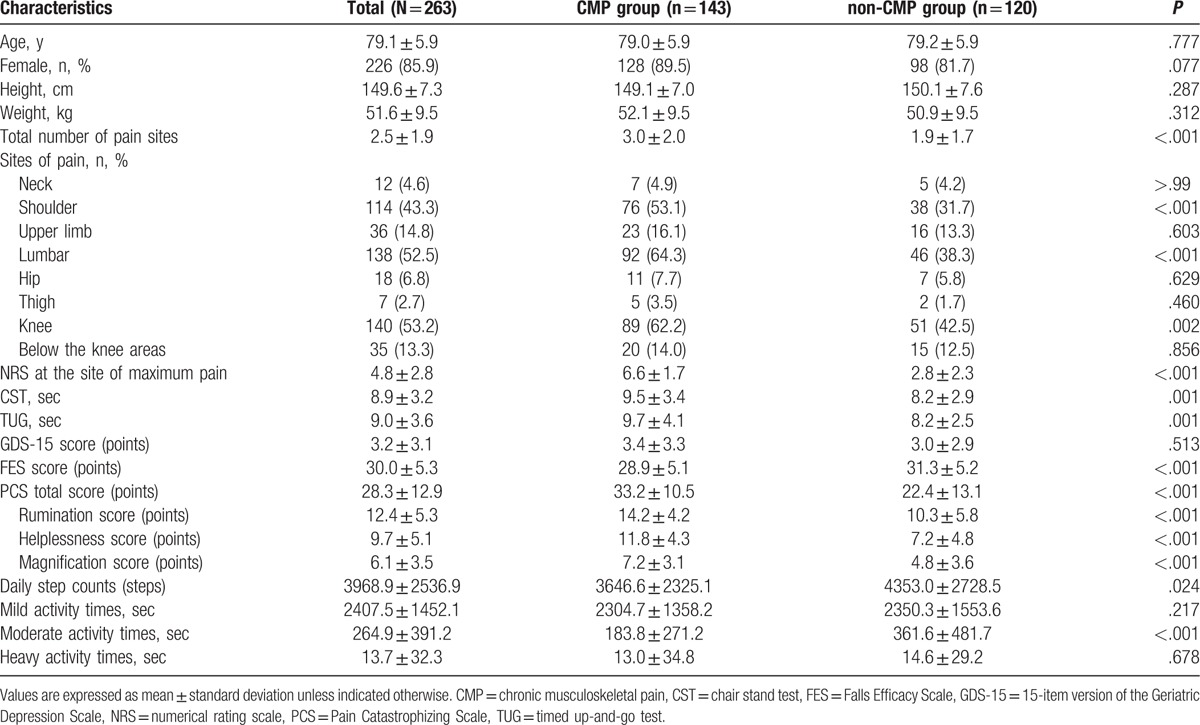
Participant characteristics and comparisons of physical function, psychological status, and physical activity between chronic musculoskeletal pain and nonchronic musculoskeletal pain groups.

### 3.2 Comparisons of physical functioning, psychological status, and physical activity between CMP and non-CMP groups

Comparisons of physical functioning, psychological status, and physical activity between the CMP and non-CMP groups are shown in Table 1. The number of participants in the CMP and non-CMP groups were 143 (54.4%) and 120 (45.6%), respectively. Regarding the pain sites in the CMP group, lumbar pain (64.3%) exhibited the highest incidence followed by the knee (62.2%) and shoulder (53.1%) regions. There were no significant between-group differences in age, sex, height, and weight. Additionally, the mean CMP group CST and TUG values were significantly higher than those in the non-CMP group.

The mean CMP group FES scores were significantly lower than in the non-CMP group. In addition, the mean CMP group values for the total PCS, rumination, helplessness, and magnification scores were significantly higher than those in the non-CMP group. There was no significant between-group difference in mean GDS-15 scores.

The mean CMP group daily step count values and moderate activity times were significantly lower than those in the non-CMP group. There were no significant between-group differences in mild and heavy activity times.

### 3.3 Factors most strongly associated with the presence of CMP

The results of the logistic regression analysis are shown in Table 2. Analyses were conducted with CMP presence as the dependent variable. Items demonstrating significant differences in previous between-group comparisons were designated as the independent variables. We evaluated multicollinearity among the independent variables using Pearson correlation coefficient. There were strong correlations between the total PCS score and each of the 3 PCS domain scores (*r* > 0.80). Additionally, there was a strong correlation between the PCS helplessness and magnification scores (*r* > 0.80). Therefore, the PCS total and magnification scores were excluded as independent variables. Based on this analysis, PCS helplessness score was identified as a significant factor associated with the presence of CMP.

**Table 2:**
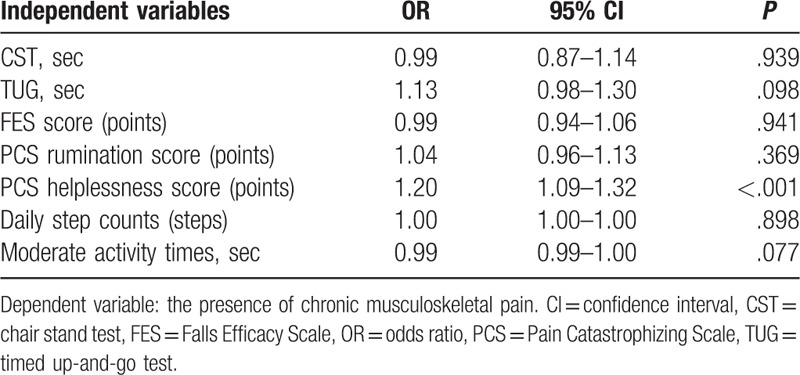
Logistic regression analysis identifying the factors most strongly associated with the presence of chronic musculoskeletal pain.

### 3.4 Estimated values of factors most strongly associated with the presence of CMP

**Figure 1:**
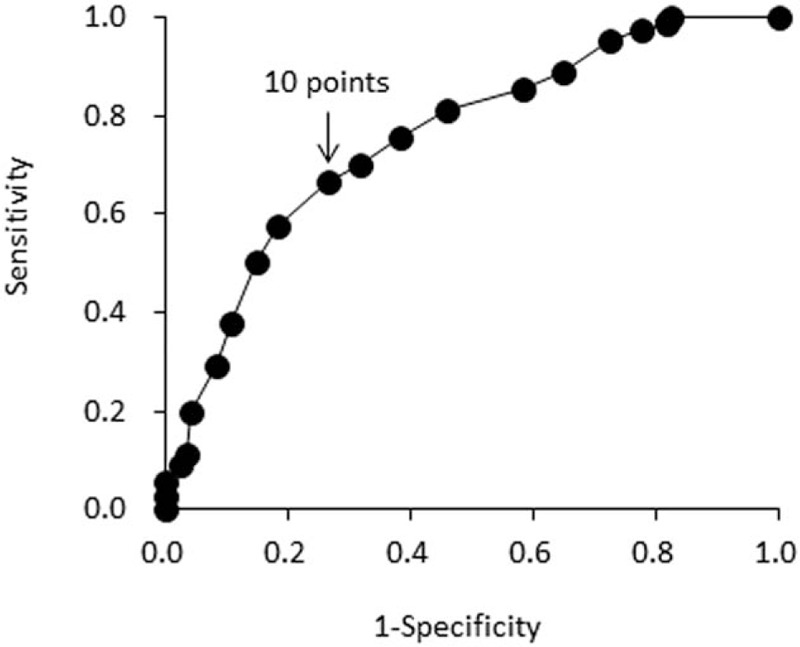
Receiver operating characteristic curve of the Pain Catastrophizing Scale helplessness scale for estimating the presence of chronic musculoskeletal pain. The area under the curve value is 0.76 (*P* < .001, 95% confidence interval: 0.70–0.81).

The results of ROC analysis are shown in Fig. 1. The area under the ROC curve of the PCS helplessness was 0.76 for estimating the presence of CMP. The best cut-off point was 10 points, with sensitivity and specificity of 66.4% and 73.3%, respectively.

## 4 Discussion

In this study, we investigated the characteristics of older adults with CMP who participated in community exercise classes. Additionally, we identified the factors that were most strongly associated with the presence of CMP in terms of physical functioning, psychological status, and physical activity level. CMP among community-dwelling older adults is a growing concern in Japan because of the rapidly aging population. However, effective treatments and prevention strategies for older adults with CMP have yet to be developed because there is a lack of recognition that CMP is a serious social issue in Japan.^[1,14]^ Consequently, this study is both novel and important because it is the first to examine the factors that are most strongly related to CMP among the Japanese population.

Regarding the number of Japanese community-dwelling older adults with CMP, only 2 studies had investigated the prevalence of CMP using the same definition in this research by postal surveys.^[1,14]^ The former reported that 223 (11.8%) of 1890 community-dwelling older adults aged 70 to 79 years had CMP; the latter reported that 111 (21.9%) of 506 community-dwelling older adults aged 71 to 80 years had CMP. In the current study, to investigate the prevalence of CMP, pain assessment was conducted by physical therapists who received training in the assessment protocols, and 143 (54.4%) of the 263 participants had CMP, suggesting a high prevalence compared to these previous studies. Therefore, our data are credible since the pain assessment was conducted by trained physical therapists and suggests that early identification of community-dwelling older adults with CMP and implementation of an effective intervention for them is urgently needed because many older adults in the community experience CMP.

In the present study, lumbar pain (64.3%) had the highest incidence followed by the knee (62.2%) and shoulder (53.1%) regions as the pain sites in the CMP group. Kitayuguchi et al^[24]^ reported that 292 (59.5%) of 491 community-dwelling older adults had lower back pain. Patel et al^[25]^ indicated that lumbar pain exhibited the highest incidence followed by the knee and shoulder regions. Therefore, an assessment of lumbar pain is important for community-dwelling older adults with CMP because the pain sites were similar to the results of previous studies.

In the current study, physical functioning performance, as determined by the CST and TUG tests, and physical activity levels such as daily step counts and moderate activity times in the non-CMP group, was significantly better than those of the CMP group. Moreover, fear of falling and pain-related catastrophizing in the CMP group were significantly higher than in the non-CMP group. Previous studies have consistently reported that the CST and TUG test values in older adults without CMP were significantly better than those for individuals with CMP.^[26,27]^ Likewise, Stubbs et al’^[27]^ systematic review and meta-analysis revealed that older adults with CMP exhibited significantly lower physical activity levels compared to those without CMP.^[28]^ Additionally, CMP was reportedly related to reinforcement of fear of falling^[27]^ and pain-related catastrophizing in older adults.^[29]^ Therefore, it appears that CMP is associated with physical deterioration, poor psychological status, and low physical activity level for older adults who participate in community exercise classes.

To our knowledge, no previous studies have investigated the factors associated with the presence of CMP and the estimated value to identify community-dwelling older adults with CMP. Based on our logistic regression analysis, PCS helplessness score was the most significant factor associated with the presence of CMP. Pain medicine research has reported that pain-related catastrophizing was an important factor in the development of CMP,^[17,30]^ disability,^[31,32]^ depression,^[29,33]^ and disuse.^[17]^ Similarly, previous studies in this field showed that pain-related catastrophizing among patients with CMP is reinforced as the result of their experience with pain, as this leads to fear and activity avoidance as well as disability, depression, and disuse.^[17,34]^ Moreover, Iwaki et al^[22]^ reported that the PCS helplessness scale was most closely associated with pain intensity among patients with chronic pain. Therefore, like our findings, researchers suggest that helplessness of pain-related catastrophizing is the factor that is most strongly associated with the presence of CMP for community-dwelling older adults. In the present study, the area under the ROC curve for the PCS helplessness scale was 0.76 for the estimation of identification of older adults with CMP, and the best cut-off point was 10 points, with sensitivity and specificity of 66.4% and 73.3%, respectively. Consequently, our findings suggest that an assessment of the PCS helplessness scale with an estimated value of 10 points is important for early identification of older adults with CMP. In practice, intervention programs that aim to improve factors associated with the PCS helplessness domain by modifying the cognitive aspects of pain may be a beneficial means of managing CMP in Japanese community-dwelling older adults.

There are several limitations to this study. First, participants actively attended the community exercise classes; therefore, it is possible that they were already health conscious and could walk by themselves. Consequently, they could be classified as highly independent older adults. Therefore, our findings might not be generalizable to less independent or health conscious older adults. Second, a cross-sectional design was used and could not prospectively delineate the factors associated with the occurrence of CMP. To account for this, future prospective studies are needed to investigate the factors associated with the occurrence of CMP in community-dwelling older adults. These studies would be valuable for the development of an effective intervention for individuals with CMP in this population.

## 5 Conclusions

Because of the high prevalence of older adults experiencing CMP, there is an urgent need to develop a means of preventing CMP within this population. However, at present, methods of CMP prevention for community-dwelling older adults have yet to be developed. Therefore, our findings are important because they contribute both knowledge about the early identification of these individuals as well as suggestions about effective interventions for older adults with CMP.

Specifically, we suggest that the assessment of helplessness of pain-related catastrophizing with an estimated value of 10 points is important for early identification of older adults with CMP. Furthermore, intervention programs that aim to improve factors associated with the PCS helplessness domain by modifying the cognitive aspects of pain may be beneficial. In practice, this type of intervention could reduce pain intensity, lead to healthier physical and psychological functioning, and increase physical activity levels for older adults with CMP.

## Acknowledgments

The authors thank Mr Tatsuya Jinnouchi, Mr Kengo Shibahara, Mr Shohei Sakai, Mr Tomohide Matsumoto, and Miss Kanami Sakuta for their contributions to data collection.
